# Sexual and reproductive health of adolescents and young people: identification of demands and experiences based on a qualitative study in communities in five Brazilian cities

**DOI:** 10.1590/0102-311XEN047824

**Published:** 2025-05-19

**Authors:** Simone Souza Monteiro, Andréa Fachel Leal, Regina Maria Barbosa, Laio Magno, André Luiz Machado das Neves, Isabelle Brambilla Honorato, Daniela Riva Knauth

**Affiliations:** 1 Instituto Oswaldo Cruz, Fundação Oswaldo Cruz, Rio de Janeiro, Brasil.; 2 Instituto de Filosofia e Ciências Humanas, Universidade Federal do Rio Grande do Sul, Porto Alegre, Brasil.; 3 Núcleo de Estudos de População, Universidade Estadual de Campinas, Campinas, Brasil.; 4 Departamento de Ciências da Vida, Universidade do Estado da Bahia, Salvador, Brasil.; 5 Instituto de Saúde Coletiva, Universidade Federal da Bahia, Salvador, Brasil.; 6 Universidade do Estado do Amazonas, Manaus, Brasil.; 7 Faculdade de Medicina, Universidade Federal do Rio Grande do Sul, Porto Alegre, Brasil.

**Keywords:** Sexual Health, Youths, HIV, Reproductive Health, Prevention, Salud Sexual, Jóvenes, VIH, Salud Reproductiva, Prevención

## Abstract

This article analyzes the experiences and demands of young people regarding sexual and reproductive health. This is a qualitative study that used ethnographic observation, semi-structured interviews, and focus groups. It assessed 139 male and female participants aged 15 to 24 years living in five low-income Brazilian communities located in Porto Alegre, São Paulo, Rio de Janeiro, Manaus, and Salvador. When analyzing the experiences and demands of young people regarding sexual and reproductive health, including HIV testing, despite regional specificities, the interviewees share similar perspectives regarding the use of local public health services. Complaints about long waiting times for care and insufficient professionals were emphasized by the interviewees. At an individual level, young people have trouble accessing health information and services due to barriers such as poor knowledge about their rights, shame, and stigma associated with sexual and reproductive health issues, as well as fear of testing positive for HIV. The lack of dialogue and guidance on sexuality and health in the family also contributes to this vulnerability. At a programmatic level, discrepancies between the working conditions and dynamics of health units and the demands of adolescents and young people, associated with poor preparation of health professionals to address specific issues of this population, are highlighted as obstacles. This study concluded that there is a lack of regular spaces for learning and dialogue about sexuality, prevention, and care for the youth due to absent investment in public health care and professional training.

## Abstract (es)

Este artículo analiza las experiencias y demandas de la juventud relacionadas con la salud sexual y reproductiva. Se trata de un estudio cualitativo en el que se utilizaron las técnicas de observación etnográfica, la entrevista semiestructurada y el grupo focal. En esta investigación participaron 139 hombres y mujeres, de entre 15 y 24 años de edad, que viven en cinco comunidades brasileñas de bajos ingresos ubicadas en Porto Alegre, São Paulo, Río de Janeiro, Manaos y Salvador. El análisis de las experiencias y las demandas de la juventud relacionadas con la salud sexual y reproductiva, incluidas las pruebas del VIH, permite constatar que los y las encuestados/as comparten perspectivas similares con respecto al uso de los servicios de salud pública locales a pesar de las especificidades regionales. Predominan las quejas por retraso en la atención y falta de profesionales. Los obstáculos a que los jóvenes enfrentan individualmente para acceder a la información y los servicios sanitarios están relacionados con la falta de conocimiento sobre sus derechos, la vergüenza y el estigma asociado con los problemas de salud sexual y reproductiva, así como el temor a un resultado positivo de la prueba del VIH. La falta de diálogo y de orientación sobre sexualidad y salud en la familia también contribuye a esta vulnerabilidad. Los obstáculos en el ámbito programático están relacionados con el desajuste entre las condiciones y dinámicas laborales de las unidades sanitarias y las demandas de los adolescentes y jóvenes, así como la falta de preparación de los profesionales de la salud para abordar temas específicos de esta población. Se concluye que faltan espacios regulares de aprendizaje y diálogo sobre sexualidad, prevención y atención a la juventud, debido a la falta de inversiones en salud pública y en formación profesional.

## Introduction

Historically, the Adolescent Health Program (PROSAD, acronym in Portuguese) created in 1989 represented a milestone in terms of public health policies for the youth. However, the process of program implementation made little progress. In the following decades, important improvements were seen in recognizing the child and youth population as subjects of rights and not as objects of guardianship of the State, family or society, leading to the enactment of the Child and Adolescent Statute (ECA, acronym in Portuguese) in 1990 and the creation of the Brazilian National Youth Secretariat and the Brazilian National Council of Youth in 2005 ^1^, among other initiatives, such as the inclusion of sexual education in the Law of Guidelines and Bases of National Education in 1996 with guidelines to address sexuality in schools ^2^; the Adolescence, Contraception and Ethics Forum with discussions of the right to privacy and confidentiality of adolescents in medical consultations and recommendation of emergency contraception in situations of exposure to the risk of pregnancy ^3^; as well as actions of nongovernmental organizations (NGOs) in mobilizing and training young people in sexual and reproductive health.

Another milestone is the proposal of the Brazilian National Policy for Comprehensive Health Care for Adolescents and Young People (PNAISAJ, acronym in Portuguese) for health promotion actions, including sexuality and reproduction, based on dialogic and emancipatory approaches and intersectoral actions. This policy addressed aspects related to sexual and reproductive health, the need for educational actions, and the participation of adolescents as multipliers ^4^. However, it still has limited reach due to the lack of investment in its implementation ^5^ ^,^ ^6^.

An exploratory search in the national literature shows limited and insufficient actions of public health services for the youth population in the field of prevention and care of sexual and reproductive health. In general, services, especially primary health care, tend to provide condoms; some contraceptive methods (for example, pills and injectable contraceptives); HIV testing; testing for other sexually transmitted infections (STIs), such as syphilis and viral hepatitis; and prenatal care. There are fewer participatory activities for health promotion and education related to sexuality, reproduction and rights, as well as fewer actions to welcome and listen to the demands of young people regarding the exercise of sexuality by health professionals ^7^ ^,^ ^8^ ^,^ ^9^. Also, there is a lack of investment in the education and training of professionals and in the structure of public health services ^10^ ^,^ ^11^ ^,^ ^12^. The weakening of the work process of multidisciplinary teams, including prevention and health promotion actions, can be attributed to the underfunding of the Brazilian National Unified Health System (SUS, acronym in Portuguese). In this regard, *Constitutional Amendment n. 95/2016* limited government spending.

The scenario described was further compromised by the dismantling of policies of sexual and reproductive health and human rights during the Federal Government between 2019 and 2022, added to the serious impacts resulting from the COVID-19 pandemic, which began in 2020 and was aggravated with the mistaken responses of the Federal Government ^13^ ^,^ ^14^. In this context, Brazil presents alarming data such as cases of abortion among women under 20 years old ^15^, an increase in AIDS cases among men aged 15 to 19 years ^16^, and the high prevalence of HIV ^17^ and other STIs ^18^ among men who have sex with men (MSM) in this age group. In addition, an increase in cases of acquired, gestational, and congenital syphilis has been observed in the country ^19^, with low rates of testing for syphilis and HIV among adolescents and young people ^20^.

According to Ayres, it is impossible to analyze people’s behaviors and propose health actions not considering an understanding of “*concretely experienced interactions, always permeated by power relations, institutional structures, and relevant cultural issues...*” ^21^ (p. 199). In this sense, this study aims to identify the conditions of vulnerability regarding the access to sexual and reproductive health service among young people at the individual, programmatic, and social levels, and understand how these conditions are articulated in the daily life.

This study analyzes the experiences and demands of young people related to sexual and reproductive health, including HIV testing, using a qualitative approach in five low-income communities located in Porto Alegre (Rio Grande do Sul State), São Paulo, Rio de Janeiro, Manaus (Amazonas State), and Salvador (Bahia State). The study considered the history of each location, the mapping of community public facilities and social projects for young people, as well as social spaces and networks for the youth. The knowledge and practices of prevention and care of HIV/other STIs and reproduction was also assessed of all interviewees (139 male and female participants aged 15 to 24 years, living in the five communities).

The proposed approach is based on a socio-anthropological perspective to understand the dimensions of vulnerability related to sexual and reproductive health among young people. This focus can reveal the reach, limits, and challenges of national health policies at the local level, supporting the formulation and implementation of health actions ^22^.

## Methodological procedures

The study is part of the project *Contexts of Vulnerability to HIV among Low-income Youth: A Multicenter Study in Five Cities in Brazil*. Using a qualitative approach to understand the complex social and cultural meaning of a specific territory ^23^ ^,^ ^24^, the study aims to obtain data for the development of sexual and reproductive health interventions using the same data collection techniques in the five communities.

The first stage referred to ethnographic observation in order to map areas of social interaction of young people in leisure activities (for example, funk parties, bars, clubs, sports), work, and daily activities (going to supermarket, school, beauty salon, among others), entered in field diaries.

The second stage involved semi-structured individual interviews about conceptions and practices regarding risks related to HIV/AIDS and other STIs, prevention strategies, and relationship with health services. Around 30 interviews were conducted with male and female participants aged 15 to 24 years from each community.

The third stage involved focus groups with adolescents and young people from the communities in order to identify intervention strategies, language, and communication channels for the youth. Based on data collected, the ideas was to identify how different social and cultural contexts affect vulnerability to HIV/other STIs.

After approval of the project by the Research Ethics Committees of the five institutions involved (Federal University of Rio Grande do Sul, State University of Campinas, Oswaldo Cruz Foundation, State University of Amazonas, and State University of Bahia; CAAE: 36495120.7.0000.5327), the study was conducted in the district of Restinga, Porto Alegre (from 2019 to 2020), in the district of Brasilândia in São Paulo (from 2019 to 2020), in the Maré community in Rio de Janeiro (from 2020 to 2021), in the district of Educandos in Manaus (2021), and in the district of Arenoso in Salvador (2021). All participants aged 18 years and older signed the Informed Consent Form (ICF), while those under 18 years of age signed the Consent Form, with the Research Ethics Committees not requiring an ICF for parents and guardians.

The study covered different phases of the COVID-19 pandemic, from its emergence (2019) to the pre-vaccination period (2020), and the vaccination phase of most population (2021). It resulted in methodological adjustments, such as virtual contacts and mobilization of local interviewers.

The study universe, described in Box 1, involved interviews with 137 low-income male and female participants aged 15 to 24 years. Most of them were between 15 and 19 years old (n = 107), female (79), and cishet (129). Eleven focus groups were created in São Paulo (2), Manaus (2), Rio (3), Porto Alegre (3), and Salvador (4).

**Box 1:** Study population.

	RIO DE JANEIRO	MANAUS	SALVADOR	PORTO ALEGRE	SÃO PAULO	TOTAL
Age group (years)						
15-19	31	16	37	5	18	107
20-24	-	2	-	10	18	30
Gender identity						
Female	14	12	21	8	24	79
Male	17	6	16	7	12	58
Sexual orientation						
Heterosexual	27	16	32	15	36	126
LGBT	4	2	5	-	-	11

The adolescents and young people participating in the study were identified through contact with local informants, health service professionals, and members of social organizations, and by approaching people while walking around in the community. The interviews and focus groups were conducted in locations agreed with the participants (social organizations, health services, homes, squares, commercial establishments), and were recorded after consent. In Manaus, some interviews were conducted digitally.

The statements were categorized according to the stages of thematic content analysis ^25^, which involves an exhaustive reading of data to identify, organize, and code the core meanings associated with the study themes, as follows: sociodemographic profile, sociability, social media, relationship with health services, exposure to and prevention of HIV and STIs, pregnancy, and neighborhood. The definition of the thematic categories and respective subcategories resulted from preexisting categories used in the interview script and others that emerged from reading the material. This process enabled the creation of a framework of categories, shared by the researchers from the five locations, which served as a basis for indexing data in NVivo software (https://lumivero.com/products/nvivo), generating a single database with all 139 interviews. The interpretation of the findings considered the study objectives, the field records, and the reflections of the academic production on the theme, as well as common aspects and specificities of the different locations.

## Results and discussion

Despite the specific characteristics of all five locations, they also present similarities like socioeconomic precariousness and armed conflicts between drug trafficking groups, militias, and security forces, which generate tension and violence in the locations. Another similar aspect is the existence of spaces for youth socialization, where meetings and affective and sexual interactions occur, such as squares, bars, street fairs, and parties in open and closed spaces (including funk dances, dance parties, *paredão* - powerful car sound systems used in street parties). All locations present basic health units (UBS, acronym in Portuguese) of the SUS, public schools, and social projects of governmental organizations and NGOs, but few sexual and reproductive health actions for young people. This analysis prioritized common aspects related to the demands and experiences of the interviewees regarding relationships with health services, prevention of HIV and STIs, pregnancy, and HIV testing.

### Health care in public services

As mentioned above, the locations had UBS, including the Family Health Strategy (FHS) and hospitals that were very close or nearby. After the team tried to contact the health departments and information systems, it was not always possible to access data about the demands and types of problems of young people treated in public services.

Observation and contact with some professionals showed structural problems, lack of human resources at the health units (for example, doctors and community health workers - CHWs), in addition to the lack of government investment at the municipal, state and/or federal levels. Some professionals reported fear of working in the study communities due to the risk of armed conflicts between police and drug traffickers. Another factor refers to the demands resulting from COVID-19, which limited services for other problems, causing new health challenges ^14^.

When asked about their motivation to go to health services, most interviewees reported poor connection due to the precariousness of the care provided, as seen in long waiting for a doctor’s appointment, inability to resolve problems, and lack of professionals. In general, they only seek local UBS in case of serious illness or acute conditions (such as high fever, fracture), as exemplified in the following statement (the names are fictitious, followed by their real age and acronym of the city where they live).

“*I’ve been to the Family Clinic, but only when I was dying... for any illness. Only when you are there, in terminal conditions,* (...) *because of the overburdened care. Or UPA too. That’s it, you will receive care if you are about to die, because before that it takes a long time, só...*” (Laura, 19 years old, Rio de Janeiro).

Another important obstacle refers to the understanding of the rules that guide the care of young people under 18 years of age by health services. Current rules allow individuals aged 13 and over to schedule appointments without the presence of parents or guardians. However, young people and their families are often unaware of this rule and many health professionals do not accept it.

In less severe cases and symptoms, the search for unconventional health care practices was reported as a result of internet searches, access to drugstore and/or recommendations from reference people (like mothers and grandmothers) and community leaders, which include treatments with natural medicines, such as roots, oils, herbs, stomach drops, and teas. This practice was mentioned in field records from Salvador and Manaus, illustrated by the following statement: “*Mom rubs my throat with a piece of cotton and andiroba, and the next day I’m fine*”, highlighting the importance of popular health practices that have been transferred through generations ^26^. In particular, when there are obstacles to conventional health services, this knowledge becomes an accessible and culturally familiar option ^27^. Also, some interviewees avoid health services due to problems with the law, resulting from their involvement with drug trafficking or even because of shame and/or trouble accessing them, as mentioned below.

### Sexual and reproductive health: experiences and demands

Regarding demands in the field of sexual and reproductive health, the statements mentioned a shortage of gynecologists and urologists in health units. Also, they had to wait a long time for care and the professionals lacked training. According to a young woman from Rio de Janeiro, during her gynecological consultation, the gynecologist made no question and only recommended the use of a contraceptive medicine. She stated that the various cases of “sexual diseases” in the community are not referred to specialized services: “*at most they advise about the use of condoms*”. Another interviewee reported the lack of encouragement for natural birth.

“*The doctors did everything they could* [to prevent a natural birth]*: ‘he must be sitting down, we have to do an ultrasound because he is sitting down, you won’t be able to give birth there’.* (...) *One day I went there for blood exams and the nurses asked me: ‘Have you scheduled the birth?’ and I answered: ‘No, it will be a natural birth’. They said: ‘Oh, you still haven’t given up? Some people like to take risks and kill children’.* (...) *I don’t feel welcomed there, no. They are not prepared, and they don’t provide information*” (Thariny, 24 years old, São Paulo).

The field records showed professionals and informants reporting a high rate of STIs and pregnant young girls and limited health care. With complaints of the public service, young people who can afford, use private health services.

Although they claim that health services do not disclose information about STIs and AIDS, during the focus group in Rio de Janeiro, the participants said that health professionals were more suitable and trustful to handle these issues, without “*that judgmental look*”. In São Paulo, some reported going to UBS when they need care or information. The statements from other communities also illustrate positive experiences with public services: “*Some are more stupid, there are always some, but others are calmer. But I feel calmer, because they know. I feel good*” (Maria, 15 years old, Manaus).

The statements suggest the need to improve health care and the provision of specific services of sexual and reproductive health for young people aiming to guarantee well-being and comprehensive health ^28^. Even in communities with more positive experiences, it is important to promote an environment of trust and alterity in health services that encourages patients to seek help and access information without fear or judgment.

In addition to the care dimension, health services are important as a space for access to contraceptive methods and HIV prevention. In all five communities, access to contraceptive methods seems to be the main reason, of a preventive nature, for young people to seek health services. The most common demand comes from women seeking injectable contraceptives and prenatal care.

In the case of male condoms, most interviewees know that they are available free of charge at health units. However, their quality is considered poor, as attested in several statements: “*That yellow thing*”; “*It smells bad, doesn’t it? It’s a hard, thick plastic*”. In addition, it “*disturbs*” a “*more enjoyable*” sexual relation. Some claim that they prefer to buy condoms at the drugstore, which are thinner and more lubricated and have a scent (chocolate, strawberry, mint), in addition to being considered “*safer*”. For Barreto ^29^, the idea of “prevention” or “care” can be influenced by personal experiences and incorporated knowledge, in which sight, taste, and smell act as a “scientific category” to identify qualities and dangers in sexual practices.

However, it is important to highlight that young people fear meeting other people they know in health units, as the demands related to contraception and prevention of STIs/HIV reveal the individual’s sexual initiation. In addition to CHWs, who often live in the same neighborhood, the possibility of exposure is potentially higher in a community where everyone knows each other. They fear being the target of gossip and malicious assumptions from friends and neighbors, as illustrated in the following statement: “*Everyone is around you watching ‘wow, he’s going to get a condom, that perverted young man’*”. It was also mentioned that those who do not have the courage to get a condom at the health unit ask their friends to do it or buy it in another neighborhood, away from the family and people they know.

Regarding prevention actions for young people, only one UBS in Salvador reported conducting activities with a focus on sexual and reproductive health for young men and women through groups that discussed healthy guidelines and empowerment. Some of these initiatives are conducted by nurses who work in the Health in Schools Program (PSE, acronym in Portuguese), promoting spaces for health education, support, listening, and production of knowledge. According to the Brazilian Ministry of Education, the PSE aims to contribute to the education of students in basic education through actions to promote health, prevent diseases and health problems, and provide health care in the territories selected by municipal education and health authorities ^30^.

In Salvador, the young people participating in the UBS activities had a better understanding of health, sexual and reproductive health and STIs, contraception, body, sexuality, and pleasure. However, there is no sign of a process that can monitor the effects of the activities performed. Anyway, the presence of Universidade do Estado da Bahia (UNEB, acronym in Portuguese) in the community, with its university extension and research actions, have generated specific activities, such as the sexual and reproductive health workshops of the *Papo Reto na Escola* [Real Talk in Schools] project and sexual and reproductive health publications for the youth ^31^.

In Porto Alegre, the interviewees mentioned the dissemination of information by “itinerant facilities” promoted by the health units, but it seems to be a specific action. In São Paulo, the UBS created discussion groups and competitions in schools, which included a question box, in which the most frequent questions from students were about pregnancy, tattoos, and drugs. However, they did not mention any evaluation processes for these activities. Also in São Paulo, some CHWs describe the youth as idle and unconcerned with health and studies. On the other hand, one CHW reports that she has contact with the girls in the neighborhood and receives them in her house to talk about everything, with no judgment. There are also young CHWs. One of them reported that one of her roles is to refer pregnant teenagers to UBS, but sometimes they “*are afraid that we will tell their parents*”. In his view, contact with the community is decreasing.

In Rio de Janeiro, a doctor from the Maré health service recognizes the lack of HIV prevention activities for young people in the health units and schools in the community. According to him, some actions were conducted in the past in the community with a focus on prevention, testing, and treatment of HIV/STIs and support by nursing and social service professionals. During the focus groups, some interviewees mentioned participation in specific actions organized by the services and visits of community agents. In the 2000s, the community had health promotion and HIV prevention actions from social projects developed in partnership with local organizations, which promoted dialogue with health professionals for support to young people and their participation in the services. However, these actions were discontinued ^32^.

Given the lack of educational resources and places to discuss topics related to sexual and reproductive health, the internet is used as a source of information and to answer questions. Some interviewees stated that they receive guidance from their mother and/or father, but it is still difficult to talk about sexuality, reproduction, and health in the family, as illustrated in the statement of a 15-year-old girl from Salvador: “*My mother has never talked to me about sex, about boys, about menstruation or anything like that. When I got my period for the first time, I thought I was going to die, a red thing coming out of me* (...) *it was at the club, I went into the bathroom and started crying, I started screaming. Then a girl who was there explained it to me that day, but my mother has never talked to me*”.

The findings of our study agree with aspects highlighted in the literature ^7^ ^,^ ^8^ ^,^ ^9^ ^,^ ^10^ ^,^ ^11^ ^,^ ^12^ related to the lack of educational actions and spaces for listening and support about sexual and reproductive health for young people in SUS health services. They also indicate the failure to meet the sexual and reproductive health demands of residents aged 15 to 19 years in low-income communities, despite the country’s community-based social actions and public policies for young people in the 2000s to promote sexual and reproductive health and AIDS prevention ^32^.

The next topic will address the issue of HIV testing in terms of investments in the provision of this service in the SUS in the last decade.

### HIV testing

Current national and global responses to HIV/AIDS have prioritized Treatment as Prevention (TasP), characterized by the expansion and encouragement of regular HIV testing to refer positive cases for treatment, as well as by the provision of pre-exposure prophylaxis (PrEP) and post-exposure prophylaxis (PEP). The benefits of these guidelines include promoting early diagnosis, care, and access to new prevention strategies. However, critical analyses indicate lack of policies to address the stigma of AIDS and the conditions of vulnerability to HIV, which have been present in historical responses to the epidemic ^33^. As the result of partnerships between the government and social movements, these responses involved encouraging condom use, counseling associated with testing, harm reduction actions, and regular campaigns. In short, there is a stronger emphasis on treatment and clinical practice than in political, sociocultural, and economic aspects of the epidemic. By analyzing data and practices of young people regarding HIV testing, we intend to identify how current guidelines have reached this population.

Field records in the five communities are consistent with studies on the lack of campaigns to encourage testing among the youth population ^34^. The statements indicate that HIV testing is not part of their routines, even among young gay and trans interviewees, who are part of the so-called key populations in the national guidelines due to the higher prevalence of HIV when compared to other groups. No significant differences were observed in relation to gender, color/race, and region.

In addition to the lack of knowledge, there is some resistance to testing, whether due to the shame related to the stigma of HIV or fear of a positive result, as expressed in the words of the young woman from Manaus: “*Seek and you will find it*”. Regarding the reasons for not taking the test, some mentioned the delay in getting results. However, the argument of lack of opportunity prevails. Others justify that they are not tested because they do not feel exposed to HIV as they “*take care of themselves*”, that is, they consider that they are using protective measures. Also, some confuse HIV testing with HPV or COVID-19 testing or are even unaware of its existence.

The few who took the HIV testing did so because of some change in their health (for example, spots on the body), pressure from family or a history of STI. It is important to highlight the concern and relief with the result. A minority related the testing to prenatal care and, occasionally, as a resource for selecting a partner.

“*...I’ve already asked* [the partner to get tested] *because of cheating. And he said the same thing: ‘Do you think I have a disease?’ Then he said he used a condom with the girl. ‘But I don’t know if you did. Until you come with the test, you can forget about it’.* (...) *If it was negative then we would continue our lives as usual, just like before* [without using a condom]” (Debora, 19 years old, São Paulo).

Regarding self-testing, lack of knowledge is also observed. In São Paulo and Porto Alegre, where the topic was highly explored, young people recognize that “*taboo*”, “*resistance*”, and “*fear of the disease*” make it difficult to get tested. According to the statements, with self-testing, individuals can get tested by themselves, without having to rely on health professionals and institutions:

“*...I believe it would be an option for us to make this commitment, we wouldn't have to make an appointment with a doctor, anything like that, we would simply do it. I believe that it would reduce* [AIDS] *a lot, because there are people who have it and don’t know it. So we, the teenagers, would definitely do this more frequently. It would be a test that you could do without any commitment, you could do it at home*” (Peterson, 22 years old, Porto Alegre).

Some of the interviewees suggested that the self-testing could be available at drugstores (it’s a place everyone goes to), making access to the diagnosis more discreet and faster. Parties and social work, health and education institutions were also mentioned, accompanied by a debate on HIV/STIs prevention. A small number of participants mentioned hospitals, squares, subway stations, and cultural facilities. Concern about confidentiality due to the stigma of AIDS was also noted.

In terms of disadvantages of self-testing, some mentioned the cost; others highlighted the lack of reliability in the accuracy of the result, comparing it to pregnancy tests purchased at drugstores. To facilitate the adherence of young people to HIV self-testing and reduce stigma, some interviewees reported the importance of guidance from health professionals on how to handle and correctly apply the test. A small number of participants mentioned the fear of not knowing how to deal with the result and preferred to take the test with a health professional.

## Final considerations

When analyzing the experiences and demands of young people regarding sexual and reproductive health, including HIV testing, despite regional specificities, the interviewees from the five regions of Brazil share similar perspectives regarding the use of local public health services. Complaints about long waiting times for care and insufficient professionals were emphasized by the interviewees, which makes it difficult to fulfill their health needs in general and sexual and reproductive health in particular. Based on that, our study identified different conditions of vulnerability at the individual, programmatic, and social levels.

At an individual level, young people have trouble accessing health information and services due to barriers such as poor knowledge about their rights, shame, and stigma associated with sexual and reproductive health issues, as well as fear of testing positive for HIV. The lack of dialogue and guidance on sexuality, reproduction, and health in the family also contributes to this vulnerability.

At a programmatic level, discrepancies between the working conditions and dynamics of health units and the demands of adolescents and young people, associated with poor preparation of health professionals to address specific issues of this population, are highlighted as obstacles, in agreement with recent studies ^35^ ^,^ ^36^. According to the statements of interviewees, they go to health units only in emergency cases or serious health problems. Issues seen as less serious are treated through self-medication, based on search on the internet, conversation with the drugstore pharmacist and/or with family members. In terms of prevention, in addition to prenatal care and immunization, the reason for young people to go to health centers is their focus on contraceptive methods, especially hormonal injections by girls. The condom quality is criticized.

Health units can offer qualified care to young people by listening to their needs, through dialogue and guidance. However, there is a lack of ongoing educational actions with a focus on sexuality, prevention, and care for the youth due to poor investments in public health care and professional training. The sexual and reproductive health activities for the youth segments mentioned are specific and limited, and have no assessment of their effects. The sexual and reproductive health information provided at schools or through social media is insufficient and tends to address the gender and human rights perspective less and less in communication strategies, which may be an effect of the national and global moral crusade against the “gender ideology”, widely publicized by the Federal Government from 2019 to 2022. This scenario became worse with the COVID-19 pandemic and the dismantling of programs in the field of rights and sexual and reproductive health.

At a social level, socioeconomic, racial and gender inequalities are notable, as well as tension and violence resulting from armed conflicts and the limited reach of existing social projects. Although most interviewees are part of the public education system, some have dropped out of school. Those who do work perform low-skilled and precarious activities. These aspects, combined with structural problems in the health service, the lack of educational programs on AIDS, and the absence of the epidemic in the national public debate increase the vulnerability of young people to HIV and other STIs.

The dimensions of individual, programmatic, and social vulnerability highlighted in our study allow a comparison to the recent reality experienced by youth segments in their territories. Besides adding to national studies, these results can help guide the implementation of public policies focused on sexual and reproductive health for low-income youth populations. Although it is not feasible to promote structural changes in the short term, it is possible to invest in the development of actions to foster learning and dialogue about sexuality, reproduction, AIDS, and other STIs in schools and health services, based on local potentialities and successful experiences observed within and outside the country ^32^ ^,^ ^37^.
