# Supplementary material for: Sexual and reproductive health of adolescents and young people: identification of demands and experiences based on a qualitative study in communities in five Brazilian cities
Source: Cad Saude Publica. 2025 May 19;41(4):e00047824. doi: 10.1590/0102-311XEN047824 (PMC12091859; doi:10.1590/0102-311XEN047824)
Supplement: Translation

# Translation: Saúde sexual e reprodutiva de adolescentes e jovens: identificação de demandas e experiências a partir de estudo qualitativo em comunidades de cinco cidades brasileiras

**DOI:** 10.1590/0102-311XPT047824

**Authors:** Simone Souza Monteiro, Andréa Fachel Leal, Regina Maria Barbosa, Laio Magno, André Luiz Machado das Neves, Isabelle Brambilla Honorato, Daniela Riva Knauth

**Affiliations:** 1 Instituto Oswaldo Cruz, Fundação Oswaldo Cruz, Rio de Janeiro, Brasil.; 2 Instituto de Filosofia e Ciências Humanas, Universidade Federal do Rio Grande do Sul, Porto Alegre, Brasil.; 3 Núcleo de Estudos de População, Universidade Estadual de Campinas, Campinas, Brasil.; 4 Departamento de Ciências da Vida, Universidade do Estado da Bahia, Salvador, Brasil.; 5 Instituto de Saúde Coletiva, Universidade Federal da Bahia, Salvador, Brasil.; 6 Universidade do Estado do Amazonas, Manaus, Brasil.; 7 Faculdade de Medicina, Universidade Federal do Rio Grande do Sul, Porto Alegre, Brasil.

**Keywords:** Saúde Sexual, Jovens, HIV, Saúde Reprodutiva, Prevenção

## Abstract

Este artigo analisa as experiências e demandas juvenis relativas à saúde sexual e reprodutiva. Trata-se de um estudo qualitativo no qual foram utilizadas as técnicas de observação etnográfica, entrevista semiestruturada e grupo focal. Integraram a pesquisa 139 homens e mulheres, de 15 a 24 anos, moradores de cinco comunidades brasileiras de baixa renda, localizadas em Porto Alegre, São Paulo, Rio de Janeiro, Manaus e Salvador. Ao analisar as experiências e demandas juvenis relativas à saúde sexual e reprodutiva, incluindo o teste de HIV, considera-se que apesar das especificidades regionais, os(as) entrevistados(as) compartilham perspectivas semelhantes em relação à utilização de serviços públicos de saúde locais. Predominam as queixas de demora no atendimento e a carência de profissionais. No âmbito individual, os jovens enfrentam dificuldades em acessar informações e serviços de saúde devido a barreiras como a falta de conhecimento sobre seus direitos, a vergonha e o estigma associados a questões de saúde sexual e reprodutiva, além do receio do resultado positivo nos testes de HIV. A falta de diálogo e orientação sobre sexualidade e saúde na família também contribuem para essa vulnerabilidade. No âmbito programático, o descompasso entre as condições e a dinâmica de trabalho das unidades de saúde e as demandas de adolescentes e jovens, somados ao despreparo dos profissionais de saúde na abordagem de questões específicas dessa população, são apontados como obstáculos. Conclui-se que faltam espaços regulares de aprendizagem e diálogo sobre sexualidade, prevenção e cuidado para o segmento juvenil, decorrente da ausência de investimentos na atenção pública em saúde e na formação profissional.

## Introdução

Historicamente, o Programa Saúde do Adolescente (PROSAD), criado em 1989, representou um marco em termos de políticas de saúde pública para o segmento juvenil. Todavia, o processo de implementação do programa pouco avançou. Nas décadas seguintes, houve ganhos importantes em relação ao reconhecimento da população infanto-juvenil como sujeitos de direitos e não como objeto de tutela do Estado, da família ou da sociedade. Essa perspectiva foi expressa na promulgação do Estatuto da Criança e do Adolescente (ECA), em 1990, e na criação da Secretaria Nacional de Juventude e do Conselho Nacional de Juventude, em 2005 ^1^, entre outras iniciativas, como a inclusão da educação sexual na Lei de Diretrizes e Bases da Educação Nacional em 1996 com orientações para abordagem da sexualidade na escola ^2^, a realização do Fórum Adolescência, Contracepção e Ética com a discussão do direito à privacidade e confidencialidade dos adolescentes nas consultas médicas e recomendação de utilização de anticoncepção de emergência em situações de exposição ao risco de gravidez ^3^, bem como a atuação de organizações não governamentais (ONG) na mobilização e formação de jovens na área da saúde sexual e reprodutiva.

Outro marco diz respeito à proposição da Política Nacional de Atenção Integral à Saúde de Adolescentes e Jovens (PNAISAJ), voltada para o fomento de ações de promoção da saúde, incluindo a sexualidade e a reprodução, a partir de abordagens dialógicas e emancipatórias e de ações intersetoriais. Nessa política, os aspectos relacionados à saúde sexual e reprodutiva, a necessidade de ações educativas e a participação dos adolescentes como multiplicadores foram abordadas ^4^. Entretanto, o PNAISAJ ainda tem alcance limitado diante da ausência de investimentos para sua implementação ^5^ ^,^ ^6^.

Uma busca exploratória da literatura nacional indica que as ações dos serviços públicos de saúde para população juvenil, no campo da prevenção e do cuidado da saúde sexual e reprodutiva, são restritas e insuficientes. Em geral, os serviços, especialmente da atenção primária à saúde, tendem a disponibilizar preservativos, alguns métodos contraceptivos (como pílula, anticoncepcional injetável), o teste do HIV e de outras infecções sexualmente transmissíveis (ISTs), como sífilis e hepatites virais e o acompanhamento do pré-natal. São mais escassas as atividades participativas de promoção e educação em saúde associadas à sexualidade, reprodução e direitos e ações de acolhimento e escuta das demandas dos jovens relativas ao exercício da sexualidade, por parte dos profissionais de saúde ^7^ ^,^ ^8^ ^,^ ^9^. Ademais, faltam investimentos na formação e capacitação dos profissionais e na estrutura dos serviços públicos de saúde ^10^ ^,^ ^11^ ^,^ ^12^. O comprometimento do processo de trabalho das equipes multiprofissionais, incluindo as ações de prevenção e promoção em saúde, pode ser atribuído ao subfinanciamento do Sistema Único de Saúde (SUS). Nesa direção, cabe citar a *Emenda Constitucional nº 95/2016*, que limitou o crescimento das despesas do Estado.

O cenário descrito foi ainda mais comprometido pelo desmonte das políticas na área saúde sexual e reprodutiva e dos direitos humanos, por parte do Governo Federal entre 2019 e 2022. Somado aos graves impactos decorrentes da pandemia de COVID-19, iniciada em 2020, igualmente agudizados pelas respostas equivocadas do então governo ^13^ ^,^ ^14^. Nesse contexto, o Brasil apresenta dados preocupantes como os casos de aborto entre mulheres abaixo dos 20 anos ^15^, o aumento de casos de aids entre homens de 15 a 19 anos ^16^, além da alta prevalência de HIV ^17^ e de outras ISTs ^18^ entre homens que fazem sexo com homens (HSH) nessa faixa etária. Apesar do aumento dos casos de sífilis adquirida, gestacional e congênita no país ^19^, a testagem para sífilis e HIV entre adolescentes e jovens é baixa ^20^.

Como afirma Ayres, não é viável analisar os comportamentos das pessoas e propor ações em saúde de forma dissociada do entendimento das “*interações concretamente vividas, atravessadas sempre por relações de poder, por estruturas institucionais, por questões culturais relevantes...*” ^21^ (p. 199). Seguindo nessa direção, este artigo objetiva identificar as condições de vulnerabilidade relacionadas ao acesso à saúde sexual e reprodutiva de segmentos juvenis, nos âmbitos individual, programático e social, e compreender de que modo elas se articulam na vida cotidiana.

Para tanto, analisa as experiências e demandas juvenis relativas à saúde sexual e reprodutiva, incluindo o teste de HIV, a partir de uma pesquisa qualitativa desenvolvida em cinco comunidades de baixa renda localizadas, respectivamente, em Porto Alegre (Rio Grande do Sul), São Paulo, Rio de Janeiro, Manaus (Amazonas) e Salvador (Bahia). O estudo contemplou a história de cada localidade, o mapeamento dos equipamentos públicos comunitários e dos projetos sociais para o público juvenil, assim como os espaços e redes de sociabilidade juvenil. Foram também investigados os conhecimentos e as práticas de prevenção e cuidado sobre HIV, demais ISTs e reprodução de 139 homens e mulheres, de 15 a 24 anos, moradores das cinco comunidades.

O recorte proposto parte da contribuição do olhar socioantropológico no entendimento das dimensões de vulnerabilidade relativas à saúde sexual e reprodutiva entre segmentos juvenis. Considera-se que tal enfoque tem a possibilidade de revelar o alcance, os limites e os desafios das políticas nacionais de saúde no âmbito local, capazes de subsidiar a formulação e implementação de ações em saúde ^22^.

## Procedimentos metodológicos

O trabalho faz parte do projeto *Contextos de Vulnerabilidade ao HIV entre Jovens de Camadas Populares: Um Estudo Multicêntrico em Cinco Cidades do Brasil*. Por meio de uma abordagem qualitativa, capaz de compreender o complexo significado social e cultural de um território específico ^23^ ^,^ ^24^, a pesquisa tem o propósito de obter dados capazes de orientar o desenvolvimento de intervenções em saúde sexual e reprodutiva. O estudo utilizou as mesmas técnicas de coleta de dados nas cinco comunidades.

A primeira se caracteriza pela observação etnográfica para mapeamento das áreas de interação social juvenil em termos de lazer (como baile funk, bares, clubes, esporte), trabalho e atividades do cotidiano (como mercado, escola, salão de beleza), registrada em diários de campo.

A segunda diz respeito a entrevistas individuais semiestruturadas acerca das concepções e práticas sobre risco relativas a HIV/aids e demais ISTs, estratégias de prevenção e relação com os serviços de saúde. Foram realizadas cerca de 30 entrevistas com homens e mulheres na faixa dos 15 aos 24 anos em cada comunidade.

A terceira etapa refere-se aos grupos focais, realizados com adolescentes e jovens das comunidades, a fim de identificar as estratégias de intervenção, linguagem e canais de comunicação adequados aos segmentos juvenis. A partir dos dados coletados, objetiva-se identificar de que forma contextos sociais e culturais distintos interferem na vulnerabilidade ao HIV e a outras ISTs.

Após aprovação do projeto pelos Comitês de Ética em Pesquisa (CEPs) das cinco instituições envolvidas (Universidade Federal do Rio Grande do Sul, Universidade Estadual de Campinas, Fundação Oswaldo Cruz, Universidade do Estado do Amazonas e Universidade do Estado da Bahia; CAAE: 36495120.7.0000.5327), a pesquisa foi realizada no bairro Restinga em Porto Alegre (de 2019 a 2020), no distrito da Brasilândia em São Paulo (de 2019 a 2020), na favela da Maré no Rio de Janeiro (de 2020 a 2021), no bairro de Educandos em Manaus (2021) e no bairro de Arenoso em Salvador (2021). Todas(os) as(os) participantes de 18 anos ou mais assinaram o Termo de Consentimento Livre e Esclarecido (TCLE), enquanto os menores de 18 anos assinaram o Termo de Assentimento Livre e Esclarecido (TALE), havendo parecer favorável do CEP para dispensa de TCLE para os pais ou responsáveis.

O estudo abarcou diferentes fases da pandemia da COVID-19, seu pré-surgimento (2019), período pandêmico pré-vacinação (2020) e fase de vacinação da maioria da população (2021). O que resultou em ajustes metodológicos, como contatos e conversas virtuais e a mobilização de entrevistadores locais.

O universo do estudo, descrito no Quadro 1, envolveu entrevistas com 137 mulheres e homens, de 15 a 24 anos, de baixa renda. A maioria na faixa de 15 a 19 (n = 107), do gênero feminino (79) e heterocisgênero (129). Os 11 grupos focais foram realizados em São Paulo (2), Manaus (2), Rio de Janeiro (3), Porto Alegre (3) e Salvador (4).

**Quadro 1:** Universo de estudo.

	RIO DE JANEIRO	MANAUS	SALVADOR	PORTO ALEGRE	SÃO PAULO	TOTAL
Faixa etária (anos)						
15-19	31	16	37	5	18	107
20-24	-	2	-	10	18	30
Identidade de gênero						
Feminino	14	12	21	8	24	79
Masculino	17	6	16	7	12	58
Orientação sexual						
Heterossexual	27	16	32	15	36	126
LGBT	4	2	5	-	-	11

A identificação dos adolescentes e jovens integrantes da pesquisa ocorreu a partir de contatos com informantes locais, profissionais dos serviços de saúde e membros de organizações sociais, assim como pela aproximação das pessoas durante a circulação na comunidade. As entrevistas de grupos focais foram feitas em locais acordados com os participantes (organizações sociais, serviços de saúde, residência, praça, estabelecimento comercial), sendo gravados após consentimento. Apenas em Manaus algumas entrevistas foram feitas por meio digital.

Os depoimentos foram categorizados com base nas etapas da análise temática de conteúdo ^25^, que envolve a leitura exaustiva dos dados, objetivando identificar, organizar e codificar os núcleos de sentido associados aos temas do estudo, quais sejam: perfil sociodemográfico, sociabilidade, redes sociais, relação com serviço de saúde, exposição e prevenção do HIV, IST, gravidez e bairro. A definição das categorias temáticas e respectivas subcategorias resultou de categorias preexistentes, utilizadas no roteiro das entrevistas e outras surgidas a partir da leitura do material. Tal processo viabilizou a criação de um quadro de categorias, compartilhado pelos(as) pesquisadores(as) das cinco localidades, que serviu de base para a indexação dos dados no software NVivo (https://lumivero.com/products/nvivo/), gerando um banco de dados único com as 139 entrevistas. Na interpretação dos achados foram considerados os objetivos do estudo, os registros de campo e as reflexões da produção acadêmica sobre o tema, bem como aspectos comuns e especificidades das diferentes localidades.

## Resultados e discussão

A despeito dos cinco locais apresentarem especificidades, existem características semelhantes quanto à precariedade socioeconômica e à ocorrência de conflitos armados entre grupos do tráfico de drogas, as milícias e as forças de segurança - geradores de tensão e violência nas localidades. Outro aspecto semelhante diz respeito à existência de espaços de sociabilidade juvenil, onde ocorrem os encontros e as interações afetivas e sexuais, como praças, bares, feiras e festas em locais abertos e fechados (como baile funk, resenhas, paredão). Em todas as localidades foram identificadas unidades básicas de saúde (UBS) do SUS, escolas públicas e projetos sociais, de organizações governamentais e ONGs, mas poucas ações voltadas para saúde sexual e reprodutiva entre jovens. Esta análise priorizou aspectos comuns relativos às demandas e experiências dos(as) entrevistados(as) sobre as relações com serviços de saúde, meios de prevenção ao HIV, ISTs, gravidez e teste de HIV.

### Cuidados em saúde nos serviços públicos

Como mencionado, as localidades contavam com UBS, incluindo a Estratégia Saúde da Família (ESF) e hospitais bem próximos ou nas imediações. Após tentativas da equipe junto às secretarias de saúde e aos sistemas de informação, nem sempre foi possível ter acesso aos dados relativos às demandas e aos tipos de agravos do segmento juvenil atendido nos respectivos serviços.

O trabalho de observação e o contato com alguns profissionais apontam que as unidades de saúde apresentavam problemas estruturais e carência de recursos humanos (como médicos e agentes comunitários de saúde - ACS). Além da falta de investimentos governamentais, no âmbito municipal, estadual e/ou federal, alguns trabalhadores reportaram o receio em atuar nas comunidades estudadas em função dos riscos dos conflitos armados entre policiais e traficantes. Outro fator, refere-se às demandas provocadas pela COVID-19, que limitou o atendimento aos demais agravos e trouxe novos desafios para atenção em saúde ^14^.

Quando indagados sobre a motivação para ir aos serviços de saúde, grande parte dos(as) entrevistados(as) relatou pouco vínculo em função da precariedade do atendimento prestado, expressa pela demora no acesso à consulta, dificuldade em resolver os problemas e carência de profissionais. Em geral, eles(elas) procuram as UBS locais apenas em caso de adoecimento grave ou condições agudas (febre alta, fratura), como exemplifica o depoimento a seguir (os nomes são fictícios, seguidos da idade e sigla da cidade de moradia).

“*Já fui na Clínica da Família, mas só quando eu estava morrendo... qualquer doença. Só quando você estava ali terminal,* (...) *por causa do atendimento sobrecarregado. Ou UPA também. Porque é isso, você vai ser realmente atendido se estiver para morrer, porque antes disso demora muito, então...*” (Laura, 19 anos, Rio de Janeiro).

Outro obstáculo importante diz respeito ao entendimento das normas que orientam o atendimento de jovens com menos de 18 anos pelos serviços de saúde. As normas atuais permitem que indivíduos a partir de 13 anos marquem consultas sem a presença de pais ou responsáveis. No entanto, com frequência os(as) jovens e seus familiares desconhecem tal fato e muitos profissionais de saúde não aceitam.

Diante de casos e sintomas menos graves, foi relatada a busca por práticas de cuidado em saúde não convencionais. Tais práticas resultam de consultas à internet, do acesso à farmácia e/ou da indicação de pessoas de referência (mães, avós) e lideranças comunitárias, que incluem o uso de tratamentos com remédios naturais, como raízes, óleos, ervas, garrafadas para estômago e chás. Esse uso foi mencionado nos registros de campo de Salvador e Manaus, ilustrado pela fala: “*mamãe esbroga* [esburga] *minha garganta com algodão com andiroba, no outro dia tô boazinha*”, apontando para a importância das práticas populares em saúde, transmitidas ao longo das gerações ^26^. Especialmente quando há obstáculos no acesso aos serviços de saúde convencionais, esses saberes tornam-se uma alternativa acessível e culturalmente familiar ^27^. Foi registrado ainda que alguns entrevistados evitam os serviços de saúde devido a problemas com a justiça, decorrente do envolvimento com tráfico, ou ainda por vergonha e/ou dificuldade de acesso, referido mais adiante.

### Saúde sexual e reprodutiva: experiências e demandas

Com relação às demandas no campo da saúde sexual e reprodutiva, os depoimentos referem que nas unidades de saúde faltam ginecologistas e urologistas. Ademais, existe demora no atendimento e a ausência de preparo dos profissionais. Segundo uma jovem do Rio de Janeiro, na sua consulta ginecológica não houve perguntas, apenas a indicação do uso de anticoncepcional. Ela afirmou que os vários casos de “doenças sexuais” na comunidade não são encaminhados para o serviço especializado: “*no máximo eles orientam sobre o uso da camisinha*”. Outra entrevistada registrou a falta de estímulo para o parto normal.

“*Os médicos faziam de tudo* [para não ter parto normal]*: ‘ele deve tá sentado, tem que fazer ultrassom porque ele tá sentando, você não vai poder parir lá’.* (...) *Uma vez eu fui colher sangue e as enfermeiras: ‘já marcou o parto?’ e eu ‘não, vai ser parto natural’. Elas ‘ah, você ainda não desistiu? Tem gente que gosta de correr riscos, de matar filho por aí’* (...) *Não me sinto nem um pouco acolhida lá, não. É um despreparo deles a falta de informação*” (Thariny, 24 anos, São Paulo).

Nos registros de campo foram assinalados relatos de profissionais e informantes acerca do alto índice de IST e de meninas gestantes e dos limites na atenção em saúde. Diante das críticas ao serviço público, aqueles(as) jovens com condições de pagar recorrem aos serviços de saúde privados populares.

Apesar de afirmarem que os serviços de saúde não divulgam informações sobre IST e aids, durante o grupo focal no Rio de Janeiro foi dito que os profissionais de saúde são pessoas mais adequadas e de confiança para tratar desses assuntos e sem “*aquele olhar de julgamento*”. Em São Paulo, alguns relatam recorrer à UBS quando precisam de cuidado ou informações. Os depoimentos nas demais comunidades também ilustram experiências positivas nos serviços públicos: “*Tem uns que são mais ignorantes, sempre tem, mas tem uns que são mais calmos. Mas eu me sinto mais tranquila, porque eles sabem. Me sinto bem*” (Maria, 15 anos, Manaus).

Os relatos sugerem a necessidade de melhorar a atenção em saúde e a oferta de serviços específicos para os jovens em relação à saúde sexual e reprodutiva, visando garantir o bem-estar e a saúde integral ^28^. Até nas comunidades com experiências mais positivas é importante promover um ambiente de confiança e alteridade nos serviços de saúde que favoreça buscar por ajuda, acesso a informações sem receios ou julgamentos.

Para além da dimensão do cuidado, os serviços de saúde assumem importância como um espaço de acesso aos métodos contraceptivos e de prevenção do HIV. Em todas as cinco comunidades, o acesso aos métodos contraceptivos parece ser a principal motivação, de caráter preventivo, para os(as) jovens buscarem os serviços de saúde. A maior demanda decorre da procura das mulheres pelo anticoncepcional injetável, além do atendimento do pré-natal.

No caso do preservativo masculino, a maioria dos(as) entrevistados(as) sabe que ele é disponibilizado gratuitamente nas unidades de saúde. Entretanto, sua qualidade é considerada ruim, como atestado em vários depoimentos: “*Aquela coisa amarela*”; “*Tem um cheiro ruim, né? É um plástico duro, grosso*”. Ademais, “*atrapalha*” uma relação sexual “*mais gostosa*”. Alguns alegam preferir comprar a camisinha na farmácia, que é mais fina e mais lubrificada e com cheiro (chocolate, morango, menta), além se ser considerada mais “*segura*”. Para Barreto ^29^ a ideia de “prevenção” ou “cuidado” pode ser atravessada por experiências pessoais e saberes incorporados, nas quais a visão, o gosto e o cheiro servem como “categoria científica” para identificar qualidades e perigos nas práticas sexuais.

Todavia, importa salientar o receio de o(a) jovem encontrar pessoas conhecidas nas unidades de saúde na medida em que as demandas relativas à contracepção e prevenção do HIV e demais ISTs revelam a iniciação sexual do sujeito. Além de que por causa dos ACS, que geralmente residem no mesmo bairro, em uma comunidade onde todos se conhecem, a possibilidade de exposição é potencialmente maior. Há o medo de ser alvo de fofocas e suposições maliciosas de amigos e vizinhos, ilustrada pela fala: “*Todo mundo tá ao seu redor olhando ‘nossa, vai pegar camisinha, olha só que jovem pervertido’*”. Foi também registrado que aqueles sem coragem de pegar camisinha no posto de saúde pedem para os amigos ou compram em outro bairro, distante dos olhares de conhecidos e familiares.

Com relação às ações de prevenção voltadas para segmentos juvenis, apenas uma UBS em Salvador relatou desenvolver atividades sobre saúde sexual e reprodutiva para homens e mulheres jovens, por meio de grupos e rodas de conversa sobre orientações saudáveis e empoderamento. Parte dessas iniciativas é realizada por enfermeiras que atuam no Programa Saúde nas Escolas (PSE), promovendo espaços de educação em saúde, acolhimento, escuta e produção de conhecimento. Segundo as diretrizes do Ministério da Educação (MEC), o PSE visa contribuir para a formação dos estudantes da Educação Básica a partir de ações de promoção da saúde, de prevenção de doenças e agravos à saúde e de atenção à saúde nos territórios pactuados entre os gestores municipais de educação e de saúde ^30^.

Ao longo do trabalho de campo em Salvador, foi observado que as(os) jovens participantes das atividades da UBS tinham maior conhecimento sobre saúde, saúde sexual e reprodutiva e IST, contracepção, corpo, sexualidade e prazer. Todavia, parece não haver processos de monitoramento capazes de indicar os efeitos das atividades desenvolvidas. De qualquer modo, a presença da Universidade do Estado da Bahia (UNEB) na comunidade, a partir de ações de extensão universitária e de pesquisas, geram atividades pontuais, como as oficinas de saúde sexual e reprodutiva do projeto Papo Reto na Escola e publicações sobre o segmento juvenil na área da saúde sexual e reprodutiva ^31^.

Em Porto Alegre, foi citada a divulgação de informações pelos “postos itinerantes”, promovidas pelas unidades de saúde; mas igualmente parece ser pontual. Em São Paulo foi registrado que a UBS realizava rodas de conversa e gincanas nas escolas, que incluíam uma caixa de perguntas, cujas dúvidas mais frequentes dos(as) estudantes eram sobre gravidez, tatuagem e drogas. Entretanto, também não foram referidos processos de avaliação dessas atividades. Ainda em São Paulo, foi observado que alguns ACS descrevem a população juvenil como ociosa e despreocupada com a saúde e com os estudos. Por outro lado, uma ACS relata manter contato com as meninas do bairro, recebendo-as em sua casa para conversar sobre tudo, sem julgar. Há também ACS jovens. Um deles relatou que uma de suas funções é encaminhar adolescentes grávidas para UBS, mas, às vezes, elas “*têm medo que contemos para os pais*”. Na sua visão, o contato com a comunidade é cada vez menor.

No Rio de Janeiro, um médico do serviço de saúde da Maré reconhece a falta de atividades de prevenção ao HIV para jovens nas unidades de saúde e escolas da comunidade. Ele lembra que já houve ações na comunidade sobre prevenção, teste e tratamento do HIV, demais ISTs e de acolhimento por parte de profissionais de enfermagem e serviço social. Durante os grupos focais, parte comentou ter participado de ações pontuais, organizadas pelos serviços e recebido a visita de agentes comunitários. Convém citar que, na década de 2000, havia na comunidade ações de promoção da saúde e prevenção do HIV desenvolvidas por projetos sociais, em parceria com organizações sociais locais, que promoviam um diálogo com profissionais de saúde, visando acolhimento e participação juvenil nos serviços. Todavia, foram descontinuadas ^32^.

Diante da carência de recursos educativos e de locais para dialogar acerca de temas no campo da saúde sexual e reprodutiva, a internet é usada como uma fonte de informação e para sanar dúvidas. Alguns entrevistados(as) afirmaram receber orientações da mãe e/ou do pai, mas prevalece a dificuldade de conversar sobre sexualidade, reprodução e saúde na família, como ilustra a fala de uma jovem de Salvador de 15 anos: “*Minha mãe nunca conversou comigo sobre sexo, sobre meninos, sobre coisa de menstruação nem nada disso. Quando eu menstruei pela primeira vez, eu pensei que eu ia morrer, um negócio vermelho saindo de dentro de mim* (...) *foi no clube, eu entrei no banheiro e comecei a chorar, comecei a gritar. Aí a menina que tava lá me explicou naquele dia, mas minha mãe não conversou comigo não*”.

Os achados descritos reiteram aspectos assinalados na literatura ^7^ ^,^ ^8^ ^,^ ^9^ ^,^ ^10^ ^,^ ^11^ ^,^ ^12^, relativos à ausência de ações educativas e espaços de escuta e acolhimento sobre saúde sexual e reprodutiva para segmentos juvenis nos serviços de saúde do SUS. Tais resultados ganham relevância ao retratar o não atendimento das demandas no campo da saúde sexual e reprodutiva de moradores(as) na faixa de 15 a 19 anos de comunidades de baixa renda, a despeito do acúmulo do país em ações sociais de base comunitária e políticas públicas para jovens nos anos 2000, voltados para a promoção de mais saúde sexual e reprodutiva e prevenção da aids ^32^.

O próximo tópico irá tratar do tema da testagem do HIV, frente aos investimentos na sua oferta no SUS na última década.

### Testagem do HIV

As atuais respostas, globais e nacional, ao HIV/aids tem priorizado o tratamento como prevenção (TcP), caracterizado pela ampliação e pelo estímulo à testagem regular de HIV, visando o encaminhamento dos casos positivos para tratamento, bem como pela oferta das profilaxias pré-exposição (PrEP) e pós-exposição (PEP) ao vírus. Essas diretrizes trazem benefícios ao incentivar o diagnóstico precoce, o cuidado e acesso às novas estratégias de prevenção. Contudo, análises críticas assinalam que esse enfoque não tem sido acompanhado de investimento em políticas de enfrentamento do estigma da aids e das condições de vulnerabilidade ao HIV, presentes nas respostas históricas à epidemia ^33^. Fruto de parcerias entre governo e movimento social, tais respostas envolviam estímulo ao uso de preservativo, aconselhamento associado ao teste, ações de redução de danos e campanhas regulares. Em suma, nota-se uma ênfase no âmbito do tratamento e da clínica e um certo apagamento dos aspectos políticos, socioculturais e econômicas da epidemia. Por meio da análise do conhecimento e das práticas dos(as) jovens sobre o teste do HIV, pretendemos identificar de que modo as atuais diretrizes têm chegado nessa população.

Os registros de campo nas cinco comunidades convergem com estudos sobre a carência de campanhas voltadas para o estímulo à testagem para população juvenil ^34^. Os depoimentos apontam que o teste do HIV não faz parte de suas rotinas, mesmo entre os jovens gays e trans entrevistados, que integram as denominadas populações-chave nas diretrizes nacionais em decorrência da maior prevalência de HIV, quando comparado com os demais segmentos. Igualmente não foram observadas diferenças significativas em relação ao gênero, à cor/raça e à região.

Além da falta de conhecimento, nota-se uma certa resistência ao teste, seja pela vergonha decorrente do estigma do HIV ou receio do resultado positivo, expresso na fala da jovem de Manaus: “*Quem procura acha*”. Quantos às razões para não realizar o teste, alguns alegaram a demora do resultado. Contudo, prevalece o argumento da falta de oportunidade. Outros(as) justificam que não se testam por não se sentirem expostos ao HIV na medida em que “*se cuidam*”, ou seja, por considerarem que utilizam medidas de proteção. Há ainda os que confundem o teste de HIV com HPV ou COVID-19 ou até desconhecem a sua existência.

Os poucos que fizeram o teste, em geral, foram motivados por alguma alteração na saúde (como manchas no corpo), pressão da família ou histórico de IST. Importa salientar a preocupação e o alívio com o resultado. Uma minoria relacionou o teste ao acompanhamento do pré-natal e, eventualmente, como um recurso na seleção do(a) parceiro(a).

“*...Já pedi* [teste ao parceiro] *por causa da traição. E ele falou a mesma coisa: ‘você tá achando que eu tô com doença?’. Aí falou que usou camisinha com a menina. ‘Mas eu não sei se você usou. Enquanto você não vir com o teste, pode esquecer’* (...) *se desse negativo aí a gente continuaria uma vida normal, como antes* [sem uso de preservativo]” (Debora, 19 anos, São Paulo).

Com relação ao autoteste também predomina o desconhecimento. Em São Paulo e Porto Alegre, onde o tema foi mais explorado, os(as) jovens reconhecem que o “*tabu*”, a “*resistência*” e o “*medo da doença*” dificultam a realização do teste. Segundo as falas, o autoteste tem a vantagem de poder ser feito pelo indivíduo, sem a dependência de profissionais de saúde e instituições:

“*...Acredito que daí seria uma forma da gente fazer esse compromisso, não teria que marcar alguma coisa com médico, coisa do tipo, a gente simplesmente faria. Acredito que diminuiria muito* [a aids]*, inclusive, porque quando vê tem pessoas que têm e não sabem. Então a gente com certeza, os adolescentes eles fariam com frequência isso. Seria um teste que você faria sem compromisso nenhum, poderia fazer em casa*” (Peterson, 22 anos, Porto Alegre).

Parte dos(as) entrevistados(as) sugeriu que o autoteste fosse disponibilizado na farmácia (“*é um lugar que todo mundo vai*”), tornando o acesso ao diagnóstico mais discreto e rápido. Foram também citadas festas e instituições de assistência social, saúde ou ensino, acompanhada de debate sobre prevenção do HIV e de demais ISTs. Uma parcela mínima citou hospitais, praças, estação de metrô e equipamentos de cultura. A preocupação com o sigilo, decorrente do estigma da aids, foi registrada.

Em termos de desvantagem do autoteste, alguns mencionaram o custo; outros pontuaram a falta de confiabilidade na precisão do resultado, fazendo um comparativo com o teste de gravidez comprado em farmácias. Para facilitar a adesão da juventude ao autoteste de HIV e diminuir o estigma, alguns depoentes indicaram a necessidade da orientação de profissionais de saúde acerca do manuseio e da correta aplicação do teste. Uma minoria assinalou o receio de não saber lidar com o resultado e preferir realizar junto com profissional de saúde.

## Considerações finais

Ao analisar as experiências e demandas juvenis relativas à saúde sexual e reprodutiva, incluindo o teste de HIV, considera-se que apesar das especificidades regionais, os(as) entrevistados(as) nas cinco regiões compartilham perspectivas semelhantes em relação à utilização de serviços públicos de saúde locais. Predominam as queixas quanto à demora no atendimento e à carência de profissionais, o que dificulta o atendimento das suas necessidades de saúde, em geral e da saúde sexual e reprodutiva, em particular. Isso permitiu identificar diversas condições de vulnerabilidade nos âmbitos individual, programático e social.

No âmbito individual, observou-se que os jovens enfrentam dificuldades em acessar informações e serviços de saúde devido a barreiras, como a falta de conhecimento sobre seus direitos, a vergonha e o estigma associados a questões de saúde sexual e reprodutiva; além do receio do resultado positivo nos testes de HIV. A falta de diálogo e orientação sobre temas associadas à sexualidade, reprodução e saúde na família igualmente contribuem para essa vulnerabilidade.

No âmbito programático, o descompasso entre as condições e a dinâmica de trabalho das unidades de saúde e as demandas de adolescentes e jovens, somado ao despreparo dos profissionais de saúde na abordagem de questões específicas dessa população, são reiteradamente apontados como obstáculos, convergindo com estudos recentes ^35^ ^,^ ^36^. Segundo os depoimentos colhidos, as unidades de saúde são acessadas apenas nos casos de emergência, diante de um problema sério de saúde. Os agravos percebidos como menos graves são tratados pela automedicação, orientados por consultas na internet, na farmácia e/ou com familiares. Em termos preventivos, além do pré-natal e casos de imunização, a motivação para ida dos(as) jovens aos postos de saúde decorre da busca por métodos contraceptivos, principalmente a injeção hormonal por parte das meninas. A qualidade do preservativo disponibilizado é criticada.

As unidades de saúde têm potencial para oferecer um atendimento qualificado aos jovens por meio da escuta de suas necessidades, do diálogo e da orientação. Todavia, faltam ações educativas continuadas sobre sexualidade, prevenção e cuidado para o segmento juvenil, decorrente da ausência de investimentos na atenção pública em saúde e na formação profissional. As atividades de saúde sexual e reprodutiva para segmentos juvenis referidas, além de pontuais e limitadas, carecem de uma avaliação sobre seus efeitos. As informações de saúde sexual e reprodutiva, fornecidas na escola ou por meio das redes sociais, são insuficientes e tendem a abordar cada vez menos a perspectiva de gênero e direitos humanos nas estratégias de comunicação, o que pode ser um efeito da cruzada moral (nacional e global) contra a “ideologia de gênero”, amplamente divulgada pelo Governo Federal de 2019 a 2022. Tal cenário se agravou com a pandemia de covid e pelo desmonte de programas na área dos direitos e da saúde sexual e reprodutiva.

No âmbito social, são notáveis as desigualdades socioeconômicas, raciais e de gênero, a tensão e violência decorrentes dos conflitos armados e o pouco alcance dos projetos sociais existentes. A despeito da maioria dos(as) entrevistados(as) integrar a rede pública de ensino, parte abandonou a escola. Aqueles que trabalham exercem atividades pouco qualificadas e precarizadas. Tais aspectos, somados aos problemas estruturais no serviço de saúde, à escassez de programas educativos sobre aids e à ausência da epidemia no debate público nacional potencializam à vulnerabilidade dos(as) jovens ao HIV e demais ISTs.

As dimensões da vulnerabilidade (individual, programática e social) assinaladas possibilitam uma aproximação com a realidade recente vivenciada por segmentos juvenis em seus territórios. Além de atualizar estudos nacionais, esses resultados permitem orientar a implementação de políticas públicas sobre saúde sexual e reprodutiva para população juvenil de baixa renda. Embora não seja viável promover mudanças estruturais a curto prazo, é possível investir no desenvolvimento de ações capazes de fomentar a aprendizagem e o diálogo sobre sexualidade, reprodução, aids e demais ISTs nas escolas e serviços de saúde, tendo por base as potencialidades locais e as experiências bem-sucedidas, realizadas dentro e fora do país ^32^ ^,^ ^37^.
